# College and Hospital Notes

**Published:** 1906-03

**Authors:** 


### COLLEGE AND HOSPITAL NOTES.

THE Medical Department of the University of Buffalo has created an orthopedic service in the College dispensary at 24 High Street. For sometime past a demand has been growing for such a dispensary service, and it is expected that much good will result from its establishment in restoring to activity deformed children, thereby enabling them to become self-supporting in the near future. The cooperation of the medical profession in making this new feature of the dispensary available to those who deserve gratuitous professional services is most desirable. Dr. Roland O. Meisenbach has been appointed to take charge of this work.

---

The New York Skin and Cancer Hospital announces that Dr. L. Duncan Bulkley will give four special lectures on the local treatment of diseases of the skin on the afternoons of March 21 and 28, and April 4 and 11, 1906. Also that Dr. William Seaman Bainbridge will give a clinical lecture on malignant and non-malignant growths, on Wednesday, April 18, in the outpatient hall of the hospital, at 4:15 p. m. These lectures are free to physicians.

---

The Alumni Association of the Medical Department of the University of Buffalo through its excutive committee is preparing a post-graduate course of lectures to continue during four days of commencement week. Due announcement in detail will be made of the schedule when fully made up. It is believed that this will prove to be one of the most important, attractive and instructive meetings ever held by the association.

